# Caregivers in anorexia nervosa: is grief underlying parental burden?

**DOI:** 10.1007/s40519-023-01530-x

**Published:** 2023-02-20

**Authors:** Jeanne Duclos, Giulia Piva, Élise Riquin, Christophe Lalanne, Dominique Meilleur, Soline Blondin, EVHAN Group, Sylvie Berthoz, Jeanne Duclos, Lama Mattar, Hélène Roux, Marie-Raphaële Thiébaud, Sarah Vibert, Tamara Hubert, Annaig Courty, Damien Ringuenet, Jean-Pierre Benoit, Corinne Blanchet, Marie-Rose Moro, Laura Bignami, Clémentine Nordon, Frédéric Rouillon, Solange Cook, Catherine Doyen, Marie-Christine Mouren, Priscille Gerardin, Sylvie Lebecq, Marc-Antoine Podlipski, Claire Gayet, Malaika Lasfar, Marc Delorme, Xavier Pommereau, Stéphanie Bioulac, Manuel Bouvard, Jennifer Carrere, Karine Doncieux, Sophie Faucher, Catherine Fayollet, Amélie Prexl, Stéphane Billard, François Lang, Virginie Mourier-Soleillant, Régine Greiner, Aurélia Gay, Guy Carrot, Sylvain Lambert, Morgane Rousselet, Ludovic Placé, Jean-Luc Venisse, Marie Bronnec, Bruno Falissard, Christophe Genolini, Christine Hassler, Jean-Marc Tréluyer, Olivier Chacornac, Maryline Delattre, Nellie Moulopo, Christelle Turuban, Christelle Auger, Solange Cook-Darzens, Nathalie Godart

**Affiliations:** 1 Univ. Lille, CNRS, UMR 9193-SCALab–Cognitive and Affective Sciences, 59000 Lille, France; 2 Département de Psychiatrie, Hôpital Saint Vincent de Paul, GHICL, 59000 Lille, France; 3 Centre Hospitalier Le Vinatier, 69678 Bron, France; 4 University Lyon 1, Villeurbanne, 69000 Lyon, France; 5 Service de Psychiatrie de l’enfant et de l’adolescent, CHU Angers, Angers, France; 6 Fondation de Santé des Etudiants de France, Paris, France; 7 Laboratory of Psychology, LPPL EA4638, University of Angers, 49045 Angers, France; 8 Mitovasc Unit, UMR CNRS 6015-INSERM, 1083 Angers, France; 9 Université Paris Cité, Paris, France; 10 Department of Psychology, Adolescence and Eating Disorders Research Laboratory, Montreal University, Montreal, QC Canada; 11 UMR 1018, CESP, INSERM, University Paris-Sud, UVSQ, University Paris-Saclay, Saclay, France; 12 UFR Des Sciences de la Santé Simone Veil, Versailles, France; 13 Department of Child and Adolescent Psychiatry, Hôpital Robert Debré, Paris, France

**Keywords:** Living grief, Burden, Caregiving, Parents, Anorexia nervosa, Gender role

## Abstract

**Purpose:**

Anorexia Nervosa (AN) is a severe chronic disorder and parents’ experience of caregiving is usually marked by emotional distress and burden. Severe chronic psychiatric disorders are known to be linked with the concept of grief. Grief has not been investigated in AN. The aim of this study was to explore parents’ and adolescents’ characteristics that may be related to parental burden and grief in AN, and the link between these two dimensions.

**Methods:**

Eighty mothers, 55 fathers and their adolescents (*N*  =  84) hospitalized for AN participated in this study. Evaluations of clinical characteristics of the adolescent’s illness were completed, as well as self-evaluations of adolescent and parental emotional distress (anxiety, depression, alexithymia). Levels of parental burden were evaluated with the Experience of Caregiving Inventory and levels of parental grief with the Mental Illness Version of the Texas Revised Inventory of Grief.

**Results:**

Main findings indicated that the burden was higher in parents of adolescents with a more severe AN; fathers’ burden was also significantly and positively related to their own level of anxiety. Parental grief was higher when adolescents’ clinical state was more severe. Paternal grief was related to higher anxiety and depression, while maternal grief was correlated to higher alexithymia and depression. Paternal burden was explained by the father’s anxiety and grief, maternal burden by the mother’s grief and her child’s clinical state.

**Conclusion:**

Parents of adolescents suffering from AN showed high levels of burden, emotional distress and grief. These inter-related experiences should be specific targets for intervention aimed at supporting parents. Our results support the extensive literature on the need to assist fathers and mothers in their caregiving role. This in turn may improve both their mental health and their abilities as caregivers of their suffering child.

**Level of evidence:**

Level III: Evidence obtained from cohort or case-control analytic studies.

**Supplementary Information:**

The online version contains supplementary material available at 10.1007/s40519-023-01530-x.

## Introduction

Anorexia nervosa (AN) occurs most frequently during adolescence or early adulthood. It can last several years [1, 2] and parents are often involved as caregivers for many years [3]. Involving relatives in the treatment of AN is internationally recommended for the best illness outcome [4, 5]. But even when adequately involved in treatment, parents are faced with many coping difficulties related to their child’s illness, which can hinder the patient’s treatment and recovery [6] and in turn affect caregiving. Indeed it has been shown that caregivers of persons with an eating disorder (ED) suffer from feelings of burden and emotional distress, with high levels of depression and anxiety [7–10]. Alexithymia as an emotional state is probably part of this clinical picture of emotional distress and should be explored. Indeed, alexithymia is characterized by difficulties identifying feelings and differentiating between feelings and bodily sensations, difficulties communicating feelings and a concrete cognitive style focused on the external environment [11]. It is a personality trait that is relatively stable and influences emotional regulation abilities. Within the framework of parental emotional distress, it may be interesting to explore the concept of alexithymia as a way of evaluating whether parents identify/express such distress [11]. While an increasing body of literature has established that ED patients have high levels of alexithymia, the empirical literature on alexithymia in parents of ED patients is scarce and conflicting, with some studies finding high levels while others do not [12–14].

The concept of caregiver burden has been extensively studied. It can be divided into objective and subjective components [15]. Objective burden involves a disruption of family/household life owing to the patient’s illness, which is potentially verifiable and observable [16]. Subjective burden refers to subjective distress among family members. Within the field of ED, the level of parental burden has been shown to be linked to different factors. It is positively correlated with higher levels of parents’ emotional distress (anxiety and depression), greater time spent with the patient [17] and longer duration of the AN illness [18, 19]. The subjective experience of illness severity also appears to be a greater determinant of caregiver burden than objective indicators of illness severity [20]. In addition, studies have found that the feeling of burden is significantly higher in mothers compared to fathers [8, 18, 19, 21, 22].

Parents of children with chronic psychiatric disorders frequently describe a sense of “living loss”^1^ and grief^2^ [23–26] that accompanies a loved one’s mental disorder. Indeed, parents may feel they have lost their child’s former or idealized self, along with their previous relationship with him/her, and hopes for the future can be disrupted. Parents also struggle to make sense of their child’s role and their own role in the family. In adolescent AN, parents specifically report mourning the missed experience of a normal adolescent period. Fueled by their ongoing, ambiguous and nonfinite nature, these losses can sometimes lead to chronic sorrow, a sadness that remains throughout the lifespan of the parent–child relationship [25–28]. Parents of youngsters with AN, a life-threatening disease with a high risk of chronicity, are likely to experience these losses and resulting grief (possibly chronic sorrow as well), which may in turn hinder their ability to support their ill child and fully engage in life. In addition, the high ambiguity and uncertainty of these “non-death losses” and associated grief experiences can negatively impact parents’ ability to process their grief [24, 28, 29].

In families of patients with early signs of psychosis, Patterson’s team assessed the possible link between carers’ current grief and burden over a 9-month follow-up period [30]. Loss was highly correlated with subjective burden at both onset and follow-up, and it was stronger at follow-up. It appears that, during the early stage of adaptation to psychosis, appraisals of burden may reciprocate with the intensity of perceived loss, thus pointing to an immediate need for dealing with issues of loss prior to integrating psychoeducational interventions*.*

Applying ambiguous loss theory to a study of predictors of grief in parents of adults with early psychosis, Williams-Wengerd [28] found that parents’ ambiguity about their own role in their child’s life was highly interconnected with parental grief. Furthermore, parents’ experience of uncertainty forced them to engage in the paradoxical task of simultaneously holding on and letting go of their relationship with their child. Finally, there was a significant relationship between parental grief and depression. A qualitative analysis confirmed these results and further indicated that various forms of therapy (group, family or individual therapy) did not alleviate parents’ feelings of depression and grief, possibly suggesting the need for more specific grief-oriented interventions. In the more general context of mental illness or psychopathology, grief has been associated with decreases in parental well-being [31], and parents’ emotional responses have been found to have an impact on their child’s trajectory towards recovery [32]. In a recent study on caregiving of patients with dementia, a direct link between burden and grief has been shown, with high levels of grief amplifying the effect of a burden on caregiver depression [33].

To our knowledge, the grief process in parents of youngsters with AN has been addressed in only one clinical publication [26], describing a useful targeted loss and grief program for parent groups. Aside from this particular psychoeducational module, parental experiences of loss and grief have not been specifically addressed in major existing evidence-based therapeutic programs for AN, such as Family-Based Treatment (FBT) [34], Parent-Focused Treatment (PFT) [35], or Multi-Family Therapy (MFT) [36]. Even the transdiagnostic model of Emotion-Focused Family Therapy (EFFT) [37], which is tailored to coach and process a whole array of caregivers’ emotional experiences that are similar to those involved in grief (sadness, anger, fear, shame, loneliness), does not precisely do so within the loss and grief framework. Indeed, Williams-Wengerd ‘s [28] qualitative study cited above did suggest that various therapeutic formats are not effective in resolving parental grief unless they include a specific grief-oriented component. Furthermore, no quantitative or qualitative study has yet explored grief in AN, including its links to various concepts such as burden and emotional distress. For these reasons, we decided to first explore the link between parental burden and parental emotional distress (including alexithymia), and the link between parental burden and the child’s characteristics. Second, we will analyze the link between parental grief and parental emotional distress, and the link between parental grief and the child’s characteristics. In addition, the study explores whether the burden on parents can be explained by grief while taking into account parental emotional distress and the child’s clinical state, both of which are known to have an impact on the burden.

## Methods

### Procedure and ethics

Prior to inclusion in the EVHAN study (Evaluation of Hospitalization for Anorexia Nervosa),^3^ all participants were hospitalized for an acute AN episode in a French specialized treatment facility for life-threatening physical and/or mental conditions related to AN. Inpatient admission was based on the following criteria: a body mass index (BMI) below 14 and/or rapid weight loss and/or compromised vital functions, severe depression, high suicidal risk, chronic under-nutrition with low weight, and/or failure of outpatient care.

Inclusion criteria for adolescents were as follows: between 13 and 21 years of age, living with their parents before admission, at least one parent accepted to be interviewed and included in the study. Exclusion criteria for both patients and their parents included: refusal to participate, insufficient command of the French language and a potentially confounding somatic pathology (such as diabetes, Crohn’s disease or another metabolic disease).

A total of 80 mothers, 55 fathers and their adolescents hospitalized for AN (*N* = 84) participated in this study. The socio-economic status of each parent was recorded according to the Classification of Professions and Socio-Professional Categories of the French National Institute for Statistics and Economic Studies (INSEE [38]), operationalized in terms of parental professional status. The socio-demographic data and socio-economic status of the non-participating parents were not statistically different from those of participating parents.

### Participant measures

All participants’ data (parents and their child) were collected during the first 2 weeks of inpatient admission. In the following descriptions, parental and adolescent measures that do not specify their validation on a French population were translated and back-translated following standard translation procedures.

### Parental measures

Sociodemographic data included age, socio-economic and marital status of parents.

Experience of burden was assessed with the Experience Caregiving Inventory **(**ECI: [15]). The ECI is the gold standard to assess the burden and provides a global rating that is derived from how distressed parents feels regarding the patient’s illness [22]. It consists of 66 items assessed with a five-point Likert scale (0: never, 1: rarely, 2: sometimes, 3: often, 4: nearly always). The negative aspect of caregiving comprises eight subscales: difficult behaviors, negative symptoms, stigma, problems with services, effects on family, need for backup, dependency and sense of loss. The 2 subscales dealing with positive aspects of caregiving measure positive personal experiences and good relationships with the patients. The negative and positive subscale scores are summed up to yield total negative and total positive appraisal scores. The sum of the negative subscales produces a total negative aspect score also called burden, higher scores reflecting the higher experience of burden [15, 30]. For this study, we only considered the Total negative burden score (ECI- Burden).

Grief was assessed with the Mental Illness Version of the Texas Revised Inventory of Grief (MIV-TIG: [39]. The MIV-TIG is an adapted version of the Texas Revised Inventory of Grief (TRIG) [40], used to assess grief reactions to the death of a loved one. It was modified to assess grief as a result of a relative’s mental illness and the loss of that person as s/he was before the development of mental illness. The scale includes the different known expressions of grief such as persistent emotional distress, being constantly occupied with the lost person, and difficulties and unwillingness to acknowledge and accept the reality of the loss. The first eight items of this 24-item scale assess present grief (MIV-TIG -A), while the remaining 16 assess current grief (MIV-TIG -B). Overall, items are focused on the loss of aspirations and cherished hopes for the individual and intrusive memories of the individual as s/he used to be. Parents were asked to respond to the items based on a five-point Likert scale ranging from completely true (1) to completely false (5). Item scores are summed up into a total score (ranging from 16 to 80) where lower scores reflect higher grieving. We only considered the MIV-TIG B part, also called MIV-Grief.

Levels of anxiety were measured with the French-validated version [41] of the Hospital Anxiety and Depression Scale (HADS: [42]). The HADS comprises 14 self-reported items, where parents are asked to respond on a four-point Likert scale. Seven items deal with depression while the remaining 7 assess anxiety. A total score for emotional distress ranges from 0 to 42, with higher scores indicating more distress. For the purpose of this study, we only considered the total anxiety score (called HADS-anx), with higher scores reflecting higher levels of anxiety (ranging from 0 to 21, with probable anxiety symptomatology around a score of 8 [41, 42].

Levels of depression were evaluated with the French-validated version [43] of the Beck Depression Inventory (BDI: [44]. It consists of 21 items describing somatic and cognitive-affective depressive symptoms. Each item is scored on a scale from 0 to 3 reflecting participants’ feelings during the previous two weeks. Based on the 21 items, a total score indicates the level of depressive symptoms: minimal depressive symptoms (range 14–19), moderate depressive symptoms (range 20–29), severe depressive symptoms (range 30–63).

Levels of alexithymia were evaluated with the French-validated version [45] of the Bermond-Vorst Alexithymia Questionnaire (BVAQ-B: [46]. The B form consists of 20 self-reported items and includes five factors: Emotionalizing and Fantasizing make up the affective dimension of alexithymia while Identifying, Analyzing and Verbalizing make up the cognitive dimension. Ratings are provided on a 5-point Likert scale; total scores range from 20 to 100, with higher scores indicating higher alexithymia.

### Adolescent measures

We collected the following sociodemographic and clinical data among the adolescent population: gender, age, family composition, educational and/or professional levels, AN subtype, age at AN onset, AN duration, nutritional status by means of current body mass index (BMI) and minimum lifetime BMI.

Current AN diagnosis was based on the Diagnostic and Statistical Manual of Mental Disorders criteria: all patients had a DSM-5 diagnosis of AN [47]. The following BMI criteria were used: BMI < 10th percentile up to 17 years of age, and BMI < 17.5 for 17 years of age and above, as in previous studies [48, 49].

The French-validated version [50] of the self-report Eating Disorders Examination Questionnaire (EDE-Q 5.2: [51] was used to assess core features of ED psychopathology. A global score (0–6) was obtained through four subscales: Dietary Restraint, Eating Concerns, Weight Concerns, and Shape Concerns. Higher scores indicate greater ED psychopathology.

The French-validated version [52] of the Morgan-Russell Global Outcome Assessment Schedule or MR-GOAS [53] was used to assess essential clinical features of AN. It is a semi-structured interview that is widely used for that purpose. A quantitative score (0–12) is obtained through five subscales: Food intake, Menstrual state, Mental state, Psychosexual state and Socioeconomic status. The higher the score, the better the clinical state.

#### Determination of the factors impacting burden and grief

We considered factors impacting burden that have been reviewed in the literature: parental emotional distress (depression, anxiety); age of patient; age of AN onset; AN duration; gender; AN subtype [7, 10, 17, 19, 22]. Factors impacting grief were determined according to the existing literature on other psychiatric disorders: parental emotional distress (depression, anxiety) and illness severity (nutritional status by means of current BMI and minimum lifetime BMI, EDE-Q total score and MR-GOAS) [30, 33]. All these factors were taken into account for both burden and grief; for parental emotional distress, we added alexithymia.

## Data analysis

Descriptive data are provided as means (*M*) and standard deviations (*SD*) for continuous variables or counts (*n*) and percentages (frequencies) for categorical variables. Between group comparisons for mothers’ and fathers’ data (ECI-Burden; MIV-Grief; HADS-anxiety; BDI; BVAQ) were computed using paired t tests for continuous variables.

Univariate analyses were performed to examine the contribution of child and parent variables to burden and grief, using R’s correlations for continuous variables (ECI-Burden; MIV-Grief; HADS-anxiety; BDI; BVAQ; MR-GOAS; illness duration), or t test for qualitative variables (type of eating disorder: AN restrictive type (AN-R) and binge-eating/purging type (AN-BP).

Multivariate linear regressions were then performed with those variables that exhibited a significant link on the univariate analysis (95% CI = 95% Confidence Interval; R2 Nagelkerke).

Multivariate linear regression was then performed to examine the contribution of children’s (global clinical state and duration of AN) and parents’ variables (depression, anxiety, alexithymia) selected through the univariate analysis (*p-*value < 0.1), first to burden (model 1) and then to grief (model 2). Finally, a third model was performed to test the link between burden and grief, taking into account potential contributions identified in models 1 and 2. Multivariate linear regression results are presented as *R*^*2*^ and for each model and *β* and *p-value* for each variable.

As AN duration was correlated with children’s age and children’s age at onset (respectively, *r* = 0.33; *p* = 0.00. *r* = – 0.35; *p* = 0.00), only AN duration was included in the multivariate linear regression models. Likewise, since all variables qualifying AN clinical state were correlated to MR-GOAS [minimal BMI (*r* = 0.32; *p* = 0.00), current BMI (*r* = 0.28; *p* = 0.00), BDI-depression scale (*r* = – 0.39; *p* = 0.00), HADS-anxiety (r = – 0.30; *p* = 0.00), EDE-Q (*r* = 0.37; *p* = 0.00)], only MR-GOAS score was included in the multivariate models.

All statistical analyses were performed with the IBM SPSS Statistics 20 software, using two-tailed statistical tests and a fixed significance level of 5%.

## Results

### Descriptive data

#### Parental sociodemographic data

The 55 fathers had a mean age of 47.41 years (*SD* = 4.67) and the 80 mothers had a mean age of 45.81 years (*SD* = 5.49). Professional and socio-economic status were as follows: 40.40% of fathers were in the upper to high-middle class category, and 32.10% belonged to the middle-class category. Regarding mothers, 48.80% belonged to the middle-class category and 26.20% were in the lower–middle-class category.

Parental levels of emotional distress, grief and burden (in Supplementary Table 1). Mothers showed significantly higher scores of emotional distress than fathers for anxiety and depression, while fathers indicated significantly higher scores of alexithymia than mothers (*p* < 0.01). There were no significant differences between maternal and paternal scores of grief (MIV-Grief) and burden (ECI- burden).

#### Adolescents’ sociodemographic and clinical data

The majority of the patients were girls (96.4%, 81/84) with a mean age of 15.98 years (*SD* = 2.32). One-quarter of the sample (25%) was enrolled in primary education, 35.7% in secondary education (30/84), 7.2% in higher education (missing data for 31%). A minority was adopted (3) and 28.6% had parents who were separated. Close to half of them (46.40%) had one sibling, 31% had two, 9.5% had three and 13.1% were only children.

The sample had a mean age of AN onset of 14.61 years (*SD* = 2.38) [8;23], a mean illness duration of 1.84 years (SD = 1.67) [0.2;7.9], a BMI of 14.3 upon admission (SD = 1,39) [11.1;18.9] and a minimum lifetime BMI of 13.61 (SD = 1.55) [9.9;18.5]. The majority (53.6%) met criteria for restrictive type AN (AN-R); the rest of the sample met criteria for AN purging type. The MR-GOAS mean score was 4.90 (*SD* = 1.41) [2.04; 7.89], suggesting a poor clinical state.

### Univariate analysis: relationships between parental burden, parental grief, and parental emotional distress and/or child clinical state

Burden (Supplementary Table 2): For both mothers and fathers, their level of emotional distress and their child’s clinical state were both linked with their level of burden. For mothers, higher levels of anxiety (HADS-anxiety score; *p* = 0.03) and depression (BDI score; *p* = 0.01), a longer duration of their child’s illness (*p* = 0.04) and a worse child clinical state (MR-GOAS score, *p* < 0.0001) were all significantly associated with a higher maternal burden score (ECI – Burden score). The maternal level of alexithymia (BVAQ score) tended to be associated with a higher level of maternal burden (*p* = 0.07).

For fathers, a higher level of anxiety (HADS-anxiety score; *p* = 0.001) and a worse child clinical state (MR-GOAS score; tendency *p* = 0.06) were significantly related to a higher level of paternal burden (ECI score).

Grief (Supplementary Table 3): For both mothers and fathers, their level of emotional distress and their child’s clinical state were related to their level of grief. For mothers, higher levels of anxiety (HADS- anxiety score; *p* = 0.005), depression (BDI score; *p* < 0.0001) and alexithymia (BVAQ score; *p* = 0.001), and a worse clinical state for their child (MR-GOAS score; *p* = 0.004) were all significantly associated with a higher current grief score (lower MIV-Grief score). For fathers, higher levels of anxiety (HADS- anxiety score; *p* = 0.002) and depression (BDI score; *p* < 0.003), and a worse clinical state for their child (MR-GOAS score; *p* = 0.03) were significantly associated with a higher current grief score (lower MIV-Grief score).

### Multivariate analysis: Contribution of parental emotional distress and/or child’s clinical state to parental burden and grief

#### Contribution to parental burden

##### For mothers’ Model 1

On the basis of the univariate analysis, the following elements were introduced as explanatory variables: anxiety, depression, alexithymia, duration of illness and the child’s clinical state. Higher maternal burden was related to a worse child clinical state (MR-GOAS score, *β* = – 0.35, *p* = 0.00). However, it was not associated with mothers’ anxiety (HADS-anxiety score, *β* = 0.00, *p* = 0.96), depression (BDI score, *β* = 0.22, *p* = 0.21) or alexithymia (BVAQ score, *β* = 0.97, *p* = 0.40), nor with child AN duration (*β* = 0.15, *p* = 0.15). This model explained 18% of the variance (*R*^2^ = 0.18).

##### For fathers’ Model 1

We also performed a paternal model with the following explanatory variables: anxiety, depression and child clinical state. A higher paternal level of burden was significantly associated with a higher level of fathers’ anxiety (HADS-anxiety score, *β* = 0.48, *p* = 0.00) and worse child clinical state (MR-GOAS score, *β* = – 0.33, *p* = 0.00). Fathers’ level of burden was not associated with their level of depression (BDI score, *β* = 0.04, *p* = 0.71). This model explained 27% of the variance (*R*^2^ = 0.27).

#### Contribution to parental grief

##### For mothers’ Model 2

The following elements were introduced as explanatory variables, on the basis of the univariate analysis: anxiety, depression, alexithymia, and worse child’s clinical state. Higher grief was explained by higher levels of mothers’ depression (BDI score, *β* = – 0.55, *p* = 0.00) and alexithymia (BVAQ score, *β* = – 0.24, *p* = 0.01), and by children’s worse clinical state (MR-GOAS score, *β* = 0.36, *p* = 0.00), but not by mothers’ anxiety levels (HADS-anxiety score, *β* = 0.24, *p* = 0.11). This model explained 35% of the variance (*R*^2^ = 0.35).

##### For fathers’ Model 2

A similar paternal model was performed for grief, and the following explanatory variables were introduced: anxiety, depression, and worse child clinical state.

A higher paternal level of grief was explained by higher anxiety (HADS-anxiety score, *β* = – 0.36, *p* = 0.001) and depression (BDI score, *β* = – 0.25, *p* = 0.04) levels, and children’s worse clinical state (MR-GOAS score, *β* = 0.33, *p* = 0.001). This model explained 31% of the variance (*R*^2^ = 0.31).

#### Relationships between parental burden and grief

A third model was performed to test the relationship between burden and grief for mothers and then fathers, taking into account potential contributions identified in models 1 and 2.

#### For mothers’ Model 3

Higher mothers’ burden levels were explained by higher grief levels (ECI score, *β* = -0.31, *p* = 0.01) and worse child clinical state (MR-GOAS score, *β* = -0.24, *p* = 0.03).

The other variables were not significant: mothers’ anxiety (HADS-anxiety score, *β* = 0.10, *p* = 0.56), depression (BDI score, *β* = 0.03, *p* = 0.85) and alexithymia levels (BVAQ score, *β* = 0.02, *p* = 0.85), as well as children’s illness duration (*β* = 0.15, *p* = 0.14) were not linked with mothers’ levels of burden. This model explained 24% of the variance (*R*^2^ = 0.24).

#### For fathers’ Model 3

Fathers’ higher burden levels were explained by higher grief levels (ECI score, *β* = – 0.35, *p* = 0.01) and higher levels of anxiety (HADS-anxiety, *β* = 0.34, *p* = 0.01) but not by the other variables which were not significant: depression levels (BDI score, *β* = – 0.04, *p* = 0.71) and child clinical state (MR-GOAS score, *β* = – 0.21, *p* = 0.01). This model explained 34% of the variance (*R*^2^ = 0.34).

## Discussion

The aim of the study was first to explore the burden and grief among parents of adolescents suffering from AN, in association with parents’ and their children’s characteristics. Second, we explored possible relationships between burden and grief in parents of patients with AN. The main findings were that parental burden and grief are linked to both children’s worse clinical state and parental emotional distress; and burden is explained in both parents by parental grief and the child’s clinical state.

One of the strengths of this study is that it considered both mothers and fathers with regard to burden, emotional distress and grief. Indeed, fathers have tended to be omitted or underrepresented in this type of research [12, 14, 28]. It also studied fathers and mothers separately as they are known to have different emotional states and perceptions of their child [54]. Differences between genders have already been shown in depression [55], anxiety [56] and alexithymia [13, 54, 57]. Furthermore, The Lancet Psychiatry recently noted the importance of disaggregating findings by sex [58], a distinction which is already well established in the field of ED [21, 59], particularly with regard to expressed emotion and family role. Our results are in line with this literature since they suggest the presence of important gender and parental role differences which are related to the level of expressed emotional distress.

Both parents expressed more grief when their child’s clinical state was more severe. Fathers’ grief was related to their own levels of depression and anxiety, while mothers’ grief was associated with their own levels of depression and alexithymia. Levels of alexithymia were higher in fathers than in mothers; yet in mothers, this emotional state was more deeply related to their own grief, showing a more profound feeling of loss for their child. Our results on alexithymia, which indeed replicate other research conducted on families affected by ED [54], tend to confirm the normative male alexithymia hypothesis: men are supposed to show the greatest deficits in identifying and expressing those emotions that reflect a sense of vulnerability (hurt, sadness or fear) or express attachment [60]. Interestingly enough, Balottin’s team also found that this is true within parental couples, suggesting that men with alexithymic traits tend to have a more collusive relationship with women who are emotionally more reactive. This type of paternal defensive style may be exacerbated by the trauma of the daughter’s pathology, which in turn creates a vicious circle that may negatively impact the course and outcome of the ED [61]. It is important to take these gender-related family dynamics into account and try to normalize and rebalance them by helping both parents identify their own emotional style and by supporting fathers in developing better communication of their emotions.

Our results also suggest that mothers might be more affected by their child’s clinical state than by their own resulting psychological suffering, whereas fathers feel a higher burden related to their own anxiety. Finally, we found that the burden was explained by grief in both parents, by anxiety in the fathers, and by the child’s worse clinical state in the mothers.

Parental experiences of burden, distress and grief are known to generate dysfunctional responses to the child’s illness [7, 10]. Some of the relationships we identified between these three experiences may help clinicians carry out a more careful evaluation of these phenomena and provide more targeted therapeutic interventions. Support should include both parental components aimed at reinforcing the parental team and individualized components based on gender and parental role differences. Work with the parental team has already been well described in the literature [62, 63]. Individualized gender-specific support should take into account both shared parental burden and grief and the unique factors contributing to these experiences. Over the last two decades, our understanding and conceptualization of “non-death” loss and grief in chronic illness and disability have improved significantly, particularly in the field of early psychosis, severe mental illness and cognitive impairment [24, 25, 28, 29]. Yet published contributions to the ED field based on non-death loss and grief theories are very rare and to our knowledge only one publication has proposed a specific loss and grief treatment model and program for parents of AN adolescents [26]. Extrapolating from this model, as well as from Yehene et al.’s [64] and Boss et al.’s [23] models of acquired non-death interpersonal loss, the grief process in adolescent AN appears to be particularly complex. First, it involves letting go of the person as she/he was before the illness, of one’s relationship with that “old” person, of one’s former dreams and expectations regarding that person: indeed, both the illness and the child as a developing individual preclude going back to this previous person/relationship. In addition, the caregiver has to find a way to live with and accept who the ill person is now, reconciling past ideals with today’s reality. One particularly painful task for parents of adolescents with enduring AN is to accept the irreversible loss of a normal adolescent period. These two phases of therapy are well described in Frisch & Bowman’s [26] psychoeducational parent group program. Yet, loss and grief work would be incomplete without also including work on “who the person may be in the future”, allowing for constant reworking of a continuing bond between these three personal entities through new relationship inputs and the creation of ongoing bridges between the former, present and future person. Indeed, around 50% to 60% of adolescents with AN will eventually improve or fully recover. It is, therefore, crucial to maintain or promote realistic hope in caregivers regarding the future adjustment of their ill adolescent, albeit within the framework of ambiguity and uncertainty [23], and with the acknowledgment that some psychosocial developmental tasks may be missed in the process. Working on parents’ intolerance of uncertainty may be particularly useful at this stage of loss and grief work, as it has been shown that parents caring for young people with restrictive ED exhibit strong feelings of “negative uncertainty” that contribute to a sense of distress and anxiety and undermines self-confidence and self-efficacy [65]. Another helpful intervention may be to focus on the meaning and purpose of the illness for their child, and its possible (not necessarily hopeless) course [66]. For example, encouraging each parent to understand the illness as a needed constructive transition towards adulthood rather than as a useless illness could help integrate the feeling of loss within a broader and more positive timeframe. More generally, helping parents normalize the experience of ambiguous loss and grief, regain a sense of mastery and hope, develop “both/and” thinking and reconstruct their own identity, is extremely important, as proposed by Boss and her colleagues [23, 24].

Results from this research, as well as current knowledge regarding caregiver burden in AN and “living” loss and grief theories, inspire other interventions as well, which may be readily adapted to an individual, single family, multifamily or parent group therapy format. We discussed above how alexithymia may be addressed through a gender-specific lens within one of these formats, conducting systematic work on the identification and regulated expression of parental emotions. More family-oriented interventions, which are often used in family and multifamily therapy [see 62,66], also provide fruitful explorations of family and individual functioning over time, that contribute to building bridges between the past, present and future through family sculptures and drawings, and through metaphorical exploration of family members’ main style of adaptation to the ED (animal genogram) or family identity issues. In addition, interventions based on the practice of externalization will help parents work through the loss of the “old” person or recognize its ongoing presence, depending on the severity of the illness over time.

Finally, it is essential to work on parents’ emotional distress. In this respect, while fathers need specific help in decreasing their levels of anxiety, mothers’ experienced burden needs to be given priority, maintaining a systemic perspective at all times on these unique yet interrelated parental experiences. Interestingly, research suggests that fathers’ attendance in family-oriented treatment is generally poor and even tends to decrease over time [67]. Therapists, and society in general, tend to accept fathers’ absence from treatment, thus unwittingly reinforcing their marginalization and conveying to mothers the message that they are the main change agent (and perhaps the guilty one to start with). This trend is particularly marked in the field of ED and unfortunately reinforces mothers’ feeling of burden [62]. Such vicious cycles can be worked on and interrupted without blaming one or the other parent. The clinical and empirical literature offers ample evidence of the unique and very significant role fathers play in their adolescent daughter’s recovery, including how their attendance in therapy is associated with improved treatment outcomes [59, 68]. This information can be relayed to fathers to strengthen their motivation to join the family in its fight against the illness and improve their understanding of their daughter’s difficulties [63, 67, 69]. Therapists should also be made aware of this literature as they are responsible for mobilizing absent or ambivalent fathers.

## Limitations

One of the limitations of the study is that it was conducted solely in an inpatient setting, which may have biased the sample towards a more severe ED population (medically and/or psychiatrically), compared to outpatient settings. Parents of hospitalized adolescents, who could not successfully manage the ED on an outpatient basis, or managed it at home with little or no professional help, may indeed have experienced a more acute sense of distress and failure than parents who were successfully empowered as outpatient caregivers. Separation from their child may also have triggered more pronounced feelings of loss and grief, as they face the task of mourning essential family roles and relationships, and also have to relinquish hopes that the condition would be short-term and easy to overcome. Overall, careful consideration is required before applying our results to parents involved in other types of care (outpatient, day treatment, home-based treatment) and in other health services. We nevertheless want to stress that illness variables, such as duration and severity of the illness, were taken into account in the statistical analyses.

The use of a cross-sectional design also limited our interpretation of the results, notably preventing any inferences of causality concerning associations among the concepts of burden, distress, loss and grief, and other studied factors. A longitudinal design is needed to evaluate the directional relationships among these factors. It is also important to note that, when studying loss and grief, a longitudinal time frame is essential to fully understand the complexities of the grief process, which covers parental representations of the “lost” healthy child, the present ill child, and the uncertain future of the grown adolescent or young adult. Within this time frame, future longitudinal designs will help our understanding of parents’ experiences over time.

Finally, it has been shown that the use of self-report questionnaires tends to minimizes levels of the measured concept compared to direct interview techniques [14]. This may be particularly true in alexithymia for fathers [13]. Nevertheless, we did find that fathers had higher levels of alexithymia than mothers on the basis of the BVAQ-B, suggesting that the sensitivity of this instrument may have been sufficiently adequate for our research purposes.

## Directions for Future research

Replications of the present study with larger samples of both restrictive and purging EDs are needed, as well as with both inpatient and outpatient settings, allowing for clearer differentiations between ED clinical subgroups, levels of clinical severity and caregiver roles. Further investigations may also usefully include the concept of intolerance of uncertainty, as a normative process, in the exploration of caregivers’experiences of loss and grief regarding the future of the child and the family. Most importantly, this study may promote the development and implementation of specific loss and grief therapeutic programs for this clinical population, which can be carefully evaluated within specific therapeutic contexts. This research has already encouraged us to include some components of the non-death loss and grief-oriented theories in our multifamily therapy and parent group programs for adolescent AN. Much improvement is still needed.

## Strengths and limits

The clinical implications of this research are crucial as there are few empirical guidelines for therapeutic practice in gender and parental role differences with regard to AN, particularly with regard to loss and grief. However, our findings should be strengthened by more empirical evidence.

### What is already known on this subject?

Parents of adolescents with AN suffer from emotional distress and burden. While caregiver burden in AN has been extensively explored, its relationships with parental feelings of loss and grief have not, nor have gender-role differences in shaping these relationships been investigated.

### What this study adds?

Our results specifically address the experiences and interrelationships of burden, distress, loss and grief in parents of adolescents with AN, carefully analyzing both common parental components and gender-based differences between fathers and mothers. This is a first step towards a better understanding of the parental experience of non-death loss and grief in AN (and more generally in eating disorders), thus paving the way for the creation of preliminary guidelines regarding the identification and resolution of loss and grief in therapeutic contexts.

## Supplementary Information

Below is the link to the electronic supplementary material.

Supplementary file1 (DOCX 31 KB)

## Data Availability

All relevant data are within the paper. Jeanne Duclos and Nathalie Godart have full access to all the data in the study. The datasets analyzed during the current study are available from the corresponding author upon reasonable request. Christophe Lalanne takes responsibility for the integrity of the data and the accuracy of the data analysis.

## Footnotes

1 A loss that is theoretical (emotional, psychological, social) rather than marked by physical death. It is the experience of losing an important image of oneself or one’s family, of what might have been, of plans for a particular future or of expectations. Living loss (or non-death loss) can take the form of physical absence with psychological presence (ex: kidnapping, lost at sea…), or of psychological absence with physical presence (ex: chronic mental illness, dementia…). Because living loss is ambiguous, it cannot be resolved and tends to be ongoing.

2 Grief is the whole-body response to these losses, which may take a cognitive, emotional, spiritual or physical form.

3 The EVHAN study (Eudract number: 2007-A01110-53, Clinical trials) is a multicenter research project conducted in France between March 2009 and December 2012. The aims were to assess: the different treatment modalities in 11 centers in France with inpatient units specialized in eating disorders; their impact on outcome at discharge and at 1-year follow-up; and predictive factors of dropout and length of stay.
