# Correction: Caregivers in anorexia nervosa: is grief underlying parental burden?

**DOI:** 10.1007/s40519-023-01564-1

**Published:** 2023-04-19

**Authors:** Jeanne Duclos, Giulia Piva, Élise Riquin, Christophe Lalanne, Dominique Meilleur, Soline Blondin, EVHAN Group, Nathalie Godart, Sylvie Berthoz, Lama Mattar, Hélène Roux, Marie-Raphaële Thiébaud, Christophe Lalanne, Sarah Vibert, Tamara Hubert, Annaig Courty, Damien Ringuenet, Jean-Pierre Benoit, Corinne Blanchet, Marie-Rose Moro, Laura Bignami, Clémentine Nordon, Frédéric Rouillon, Solange Cook, Catherine Doyen, Marie-Christine Mouren, Priscille Gerardin, Sylvie Lebecq, Marc-Antoine Podlipski, Claire Gayet, Malaika Lasfar, Marc Delorme, Xavier Pommereau, Stéphanie Bioulac, Manuel Bouvard, Jennifer Carrere, Karine Doncieux, Sophie Faucher, Catherine Fayollet, Amélie Prexl, Stéphane Billard, François Lang, Virginie Mourier-Soleillant, Régine Greiner, Aurélia Gay, Guy Carrot, Sylvain Lambert, Morgane Rousselet, Ludovic Placé, Jean-Luc Venisse, Marie Bronnec, Bruno Falissard, Christophe Genolini, Christine Hassler, Jean-Marc Tréluyer, Olivier Chacornac, Maryline Delattre, Nellie Moulopo, Christelle Turuban, Christelle Auger, Solange Cook-Darzens, Nathalie Godart

**Affiliations:** 1 Univ. Lille, CNRS, UMR 9193-SCALab–Cognitive and Affective Sciences, 59000 Lille, France; 2 Département de Psychiatrie, Hôpital Saint Vincent de Paul, GHICL, 59000 Lille, France; 3 Centre Hospitalier Le Vinatier, 69678 Bron, France; 4 University Lyon 1, 69000 Villeurbanne, France; 5 Service de Psychiatrie de l’enfant et de l’adolescent, CHU Angers, Angers, France; 6 Fondation de Santé des Etudiants de France, Paris, France; 7 Laboratory of Psychology, LPPL EA4638, University of Angers, 49045 Angers, France; 8 Mitovasc Unit, UMR CNRS 6015-INSERM, 1083 Angers, France; 9 Université Paris Cité, Paris, France; 10 Department of Psychology, Adolescence and Eating Disorders Research Laboratory, Montreal University, Montreal, QC Canada; 11 UMR 1018, CESP, INSERM, University Paris-Sud, UVSQ, University Paris-Saclay, Saclay, France; 12 UFR Des Sciences de la Santé Simone Veil, Versailles, France; 13 Department of Child and Adolescent Psychiatry, Hôpital Robert Debré, Paris, France

**Correction:** **Eating and Weight Disorders - Studies on Anorexia, Bulimia and Obesity (2023) 28:16 ****https://doi.org/10.1007/s40519-023-01530-x**

In this article, the order that the authors appeared in the author list was incorrect [1]. This has been corrected in this correction. The original article has been corrected.
