# Supplementary material for: Caregivers in anorexia nervosa: is grief underlying parental burden?
Source: Eat Weight Disord. 2023 Feb 20;28(1):16. doi: 10.1007/s40519-023-01530-x (PMC9941225; doi:10.1007/s40519-023-01530-x)
Supplement: Supplementary file 1 — Supplementary file1 (DOCX 31 KB) [file 40519_2023_1530_MOESM1_ESM.docx]

**S. Table 1. Parental levels of emotional distress, grief and burden.**

|  | Father | | Mother | |  |
| --- | --- | --- | --- | --- | --- |
|  | *n* | *m (SD)* | *n* | *m (SD)* |  |
| HADS – anx | 54 | 6.94 (3.63) | 80 | 9.23 (4.79) | r(53)=.058, p<.0001* |
| BDI | 55 | 8.76 (6.02) | 80 | 14.88 (9.71) | r(53)=.346, p<.08 * |
| BVAQ-B total score | 55 | 54.63 (8.84) | 80 | 49.72 (9.37) | r(53)=-.109, p<.002 * |
| ECI - Burden | 55 | 124.25 (26.29) | 78 | 132.28 (24.69) | r(53)=.346, p<.08 |
| MIV - Grief | 55 | 48.18 (11.41) | 79 | 45.40 (11.29) | r(53)=.152, p<.09 |

Legends: *n* = sample; *m* = mean; *SD* = standard deviation; *HADS- anx* = Hospital Anxiety and Depression Scale – anxiety subscale; *BDI* = Beck Depression Inventory; *BVAQ* = Bermond-Vorst Alexithymia Questionnaire; *ECI* = Experience Caregiving Inventory; *MIV-Grief* = current grief; *comparison between mother and father paired t test p<0.01

**S. Table 2. Correlation between parental burden (ECI-Burden) and parental distress (HADS-anxiety, BDI, BVAQ) and patients’ clinical state (illness duration and Morgan-Russell Assessment Schedule)**

|  | ECI-Burden score Father | | | ECI-Burden score Mother | | |
| --- | --- | --- | --- | --- | --- | --- |
|  | *n* | *r* | *p* | *n* | *r* | *p* |
| Parental HADS - anx | 54 | 0.45** | 0.001 | 78 | 0.25* | 0.03 |
| Parental BDI | 54 | 0.24 | 0.09 | 78 | 0.29** | 0.01 |
| Parental BVAQ | 55 | 0.01 | 0.93 | 78 | 0.21 | 0.07 |
| Child AN Duration | 53 | 0.17 | 0.23 | 75 | 0.23* | 0.04 |
| Child MR - GOAS | 55 | -0.25 | 0.06 | 78 | -0.39** | 0.00 |

Legends: *n* = sample; *r* = Pearson’s coefficient; *p* = p-value; *HADS- anx* = Hospital Anxiety and Depression Scale – anxiety subscale; *BDI* = Beck Depression Inventory - depression; *BVAQ* = Bermond-Vorst Alexithymia Questionnaire - alexithymia; *ECI-Burden* = Experience Caregiving Inventory - burden; *MIV-Grief* = Mental Illness Version of Texas Inventory Grief – B = current grief; *MR-GOAS*: Morgan-Russell Global Outcome Assessment Score – clinical status. * The correlation is significant to the level 0,05 (bilateral) ** the correlation is significant to the level 0,001 (bilateral).

**S. Table 3: Correlation between level of parental current grief (MIV-TIG-B) and parental distress (HADS-anxiety, BDI, BVAQ), patients’ clinical state (illness duration and Morgan-Russell Assessment Schedule)**

|  | MIV-Grief Father | | | MIV-Grief Mother | | |
| --- | --- | --- | --- | --- | --- | --- |
|  | *n* | *r* | *p* | *n* | *r* | *p* |
| Parental HADS - Anxiety | 54 | -0.4^**^ | 0.002 | 79 | -0.3^**^ | 0.005 |
| Parental BDI - Depression | 54 | -0.4^**^ | 0.003 | 79 | -0.5^**^ | 0.00 |
| Parental BVAQ - Alexithymia | 55 | -0.04 | 0.8 | 79 | -0.4^**^ | 0.001 |
| Child AN Duration | 53 | 0.04 | 0.8 | 76 | -0.1 | 0.41 |
| Child MR - GOAS | 55 | 0.3 | 0.03 | 79 | 0.3^**^ | 0.004 |

Legends: *n* = sample; *r* = Pearson’s coefficient; *p* = p-value; *HADS* = Hospital Anxiety and Depression Scale; *BDI* = Beck Depression Inventory; *BVAQ* = Bermond-Vorst Alexithymia Questionnaire; *MR-GOAS*: Morgan-Russell Global Outcome Assessment Score, * the correlation is significant to the level 0,05 (bilateral) ** the correlation is significant to the level 0,001 (bilateral)
